# Catestatin and Advanced Glycation End-Products: Potential Indicators of Cardiovascular Risk in Hashimoto’s Thyroiditis

**DOI:** 10.3390/biom15020169

**Published:** 2025-01-23

**Authors:** Petra Punda, Marko Kumric, Ana Baric Zizic, Sanda Sladic, Marko Vuletic, Daniela Supe Domic, Marino Vilovic, Doris Rusic, Josko Bozic

**Affiliations:** 1 Clinical Department of Diagnostic and Interventional Radiology, University Hospital of Split, Spinciceva 1, 21000 Split, Croatia; zubanovicpetra@gmail.com; 2 Department of Pathophysiology, University of Split School of Medicine, Soltanska 2A, 21000 Split, Croatia; marko.kumric@mefst.hr (M.K.); marino.vilovic@mefst.hr (M.V.); 3 Laboratory for Cardiometabolic Research, University of Split School of Medicine, Soltanska 2A, 21000 Split, Croatia; 4 Department of Nuclear Medicine, University Hospital of Split, Spinciceva 1, 21000 Split, Croatia; abaric@kbsplit.hr (A.B.Z.); sgracan@kbsplit.hr (S.S.);; 5 Department of Nuclear Medicine, University of Split School of Medicine, Soltanska 2A, 21000 Split, Croatia; 6 Department of Health Studies, University of Split, Rudera Boskovica 35, 21000 Split, Croatia; dsupe@kbsplit.hr; 7 Department of Medical Laboratory Diagnostics, University Hospital of Split, Spinciceva 1, 21000 Split, Croatia; 8 Department of Pharmacy, University of Split School of Medicine, Soltanska 2A, 21000 Split, Croatia; doris.rusic@mefst.hr

**Keywords:** Hashimoto’s thyroiditis, advanced glycation end-products, catestatin, endothelial dysfunction, cardiovascular risk

## Abstract

Accumulating evidence suggests that overt hypothyroidism is associated with accelerated atherosclerosis, thereby increasing the risk for major cardiovascular events. The present study aimed to investigate the associations between serum catestatin levels and advanced glycation end-products (AGEs), indicators of vascular health, in individuals with Hashimoto’s thyroiditis compared to healthy controls. A total of 100 female patients with Hashimoto’s thyroiditis and 100 age-matched healthy controls were included in the study. Serum catestatin levels (10.2 (6.5–15.8) vs. 6.4 (4.1–9.3) ng/mL, *p* < 0.001) and tissue levels of AGEs (2.21 ± 0.55 ng/mL vs. 1.89 ± 0.56, *p* < 0.001) were both significantly higher in the Hashimoto’s group compared to the healthy age-matched controls. A positive correlation was observed between catestatin and AGEs in the overall population (r = 0.489, *p* < 0.001) and within the Hashimoto’s group (r = 0.457, *p* < 0.001). Additionally, weak positive correlations were noted between catestatin and high-sensitivity C-reactive protein, as well as anti-thyroid peroxidase antibodies (r = 0.277, *p* = 0.005 and r = 0.229, *p* = 0.024, respectively). All of these associations were confirmed through multivariate analyses. The present analysis indicates that catestatin might be implicated in cardiovascular consequences of Hashimoto’s thyroiditis. However, future research should focus on longitudinal studies to explore if the causal relationship exists.

## 1. Introduction

Hashimoto’s thyroiditis is one of the most prevalent autoimmune disorders currently observed [1]. Although there have been significant advancements in the early detection and management of this condition, optimal hygienic and dietary recommendations tailored to individuals with Hashimoto’s thyroiditis have yet to be fully established. Emerging research highlights the potential of dietary interventions to influence not only the incidence but also the progression of various diseases, particularly those within the autoimmune spectrum [2,3,4].

Accumulating data suggest that overt hypothyroidism is associated with accelerated atherosclerosis, thus conferring risk for the development of major cardiovascular incidents [5]. Although some of these effects can be attributed to a higher prevalence of hypertension and dyslipidemia in patients with hypothyroidism, not all individuals with hypothyroidism have abnormal blood pressure or lipid profiles. Hence, it seems that hypothyroidism confers independent cardiovascular risk [6]. The development and progression of endothelial dysfunction seems to be an important underlying mechanism. In recent years, several biomarkers emerged as indicators of endothelial function. On the other hand, catestatin, a potent physiological inhibitor of catecholamine spillover, is also implicated in the preservation of endothelial function and immune system regulation [7,8]. Advanced glycation end-products (AGEs) are harmful compounds formed through the non-enzymatic glycation of proteins, lipids, and nucleic acids, primarily in conditions of hyperglycemia [9]. Their accumulation is associated with various chronic diseases, including diabetes and cardiovascular diseases, as they can promote oxidative stress and inflammation, contributing to endothelial dysfunction [10]. In the context of Hashimoto’s thyroiditis, elevated levels of AGEs have been linked to increased oxidative stress and inflammation, which may exacerbate the disease’s progression and its associated cardiovascular risks [11].

This study aims to investigate the associations between serum catestatin levels and AGEs in individuals with Hashimoto’s thyroiditis compared to healthy controls. Specifically, we aimed to elucidate whether elevated catestatin levels are associated with hsCRP and autoantibodies, as well as to examine the potential role of catestatin in mediating cardiovascular risk in Hashimoto’s thyroiditis. By analyzing these relationships, the study aims to contribute to a better understanding of the cardiovascular implications of Hashimoto’s thyroiditis and the role of catestatin and AGEs in this context.

## 2. Materials and Methods

The present research was designed as a single-center, cross-sectional study. The study was conducted at the Department of Nuclear Medicine, University Hospital of Split in the period from February 2022 to September 2023. Participant recruitment commenced following the approval granted by the Ethics Committee of the University Hospital of Split (2181-147/01/06/M.S.-22-02). All participants provided written informed consent prior to enrollment. No compensation was offered to participants for their involvement in the study.

The inclusion criteria specified female patients, aged 18 to 60, with a recent diagnosis of Hashimoto’s thyroiditis who had not initiated thyroid hormone replacement therapy. Exclusion criteria included treatment of Hashimoto’s thyroiditis with thyroid hormone replacement therapy prior to study enrollment, arterial hypertension, history of advanced heart failure (NYHA III or IV), chronic renal failure, liver failure, diabetes, acute infectious events, significant immunocompromise, corticosteroid therapy within the last 3 months, active malignancy, other thyroid disorders, significant psychiatric illness, other autoimmune diseases aside from Hashimoto’s thyroiditis, and a history of drug or alcohol abuse.

The control group consisted of 100 healthy women without Hashimoto’s thyroiditis. Control subjects were matched with the research group by age, sex, and body mass index (BMI). Recruitment of control subjects was conducted through researchers’ colleagues, family, friends, and acquaintances. Prior to participation, all control subjects provided informed consent, and the procedures performed on the research group were also applied to the control group. The exclusion criteria for the control group were identical to those for the research group.

Physical examination and relevant items from past medical history were collected from all patients. A Seca altimeter (Birmingham, UK) was used to measure participants’ height and weight accurately. BMI was calculated by dividing the value of body mass (kg) and the squared value of height (m^2^). The waist circumference was measured at the level of the midline between the bottom line of the rib arch, in the mid-axillary line, and the tip of the iliac crests. The hip circumference was measured at the level of the largest circumference above the line joining the great trochanters of the femur. Patients were standing upright during both measurements. The waist-to-hip ratio was calculated by dividing measured waist and hip circumference. Office BP was measured according to guidelines for BP measurement, using WatchBP Home A (Microlife AG Swiss Corporation, Widnau, Switzerland).

A sample of venous blood was obtained from the forearm (cubital vein) using a sterile disposable needle and after cleansing the skin surface. This procedure was performed by a trained laboratory technician. Up to 22 mL of blood was collected. A portion of the blood was sent for immediate analysis, while the remainder was aliquoted and stored at −80 °C for subsequent analysis of catestatin. Serum catestatin concentrations were measured using an enzyme-linked immunosorbent assay (ELISA) from Phoenix Pharmaceuticals Inc. (Burlingame, CA, USA). The assay exhibited a sensitivity of 0.05 ng/mL, with a linear detection range of 0.05–0.92 ng/mL, and demonstrated 100% cross-reactivity with endogenous human catestatin. The intra-assay and inter-assay coefficients of variation were <10% and <15%, respectively. All blood samples were analyzed at a certified institutional biochemical laboratory following standard operating procedures. The biochemist performing the analysis was blinded to the participants’ group assignments.

Patients were subsequently invited to the Department of Nuclear Medicine where levels of AGEs were measured using the AGE Reader (DiagnOptics Technologies BV, Groningen, The Netherlands). Cardiovascular risk was evaluated by measuring tissue levels of advanced glycation end-products (AGEs) using the AGE Reader (DiagnOptics Technologies BV, Groningen, The Netherlands). This non-invasive device employs ultraviolet light to stimulate autofluorescence in human skin tissue, which correlates with tissue AGE levels [12]. The AGE Reader provides a rapid cardiovascular risk assessment, delivering results within 12 s. Designed for ease of use and patient comfort, the AGE Reader is a user-friendly method that has been extensively validated.

Statistical analysis has been conducted in MedCalc (version 19.1.2., MedCalc Software, Ostend, Belgium). Figures were created using Prism for Windows^®^ (version 9.4.1., GraphPad, La Jolla, CA, USA). Quantitative data were presented as either mean ± standard deviation (SD) or median and interquartile range, depending on the distribution, whereas categorical data were presented as n (%). Normality of data distribution was assessed using the Shapiro–Wilk test. Comparisons of quantitative variables were performed using Student’s *t*-test or Mann–Whitney U test, based on the normality of the data, while categorical data were analyzed with the chi-squared test. Spearman’s rank correlation analysis was used to examine the relationships between catestatin/AGE levels and various clinical and laboratory variables. Multiple linear regression analysis was used to establish an association between catestatin/AGEs and relevant clinical and laboratory parameters. Statistical significance was defined as *p* < 0.05 for all comparisons.

Sample size analysis has been conducted using data from a pilot study on 10 randomly selected subjects from the patient population. In addition, data from 10 randomly selected matched control patients was obtained. The value of serum catestatin levels, which was the main outcome of the study, was used for the calculation. With type I error of 0.05, and the power of 80%, the required sample size was 79 participants per group. Taking into consideration a potential drop-out or loss to follow-up, we increased the number of the sample to 200 participants.

## 3. Results

The present study enrolled 100 female patients with Hashimoto thyroiditis and 100 healthy, age- and sex-matched control participants. No significant differences in baseline and anthropometric characteristics were found between patients and control group (Table 1). Regarding the laboratory values, patients with Hashimoto had significantly higher serum levels of TSH (*p* = 0.017), hsCRP (*p* < 0.001), anti-TG (*p* < 0.001), anti-TPO (*p* < 0.001) and LDL-cholesterol (*p* = 0.025). On the other hand, patients with Hashimoto were shown to exhibit lower levels of fT4 (*p* = 0.027) (Table 2).

**Figure 1:**
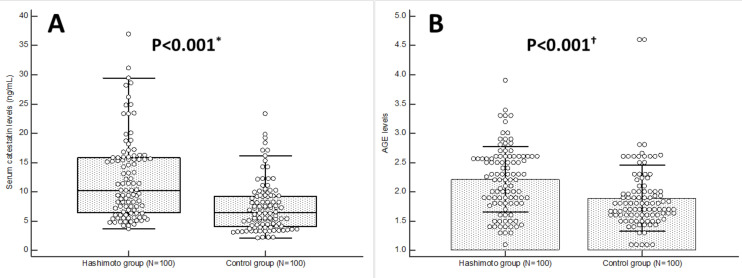
Serum catestatin levels (**A**) and AGE levels (**B**) in Hashimoto (N = 100) and control group (N = 100) participants. * Mann–Whitney test. † *t*-test for independent samples. Abbreviations: AGEs—advanced glycation end-products.

Serum catestatin levels were significantly higher in the Hashimoto group in comparison to healthy controls (10.2 (6.5–15.8) vs. 6.4 (4.1–9.3) ng/mL, *p* < 0.001) (Figure 1A). Moreover, multiple linear regression analysis has demonstrated that the presence of Hashimoto thyroiditis is significantly associated with serum catestatin levels, independent of age, BMI, systolic blood pressure, AGEs, hsCRP, TSH and anti-TPO serum concentrations (β 1.819, SE 0.830, *p* = 0.030) (Supplementary Tables S1 and S2). Tissue levels of AGEs were also significantly higher in the Hashimoto group in comparison to healthy controls (2.21 ± 0.55 ng/mL vs. 1.89 ± 0.56 ng/mL, *p* < 0.001) (Figure 1B), independent of age, BMI, systolic artery pressure, fasting glucose, serum catestatin, TSH and anti-TPO serum concentrations (β 1.419, SE 0.597, *p* = 0.019) (Supplementary Tables S3 and S4).

**Figure 2:**
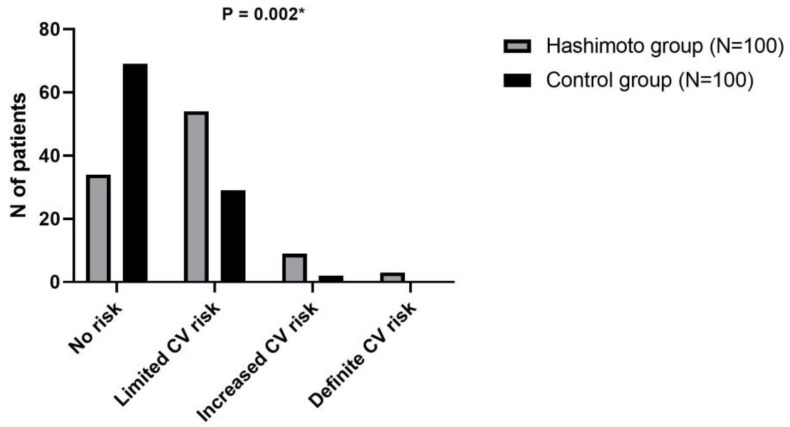
Distribution of cardiovascular risk categories according to tissue AGEs among patients with Hashimoto’s thyroiditis and control subjects. * Chi-squared test. Abbreviations: AGEs—advanced glycation end-products.

Furthermore, the distribution of cardiovascular risk categories (based on AGEs) highlights a higher prevalence of elevated risk among patients with Hashimoto’s thyroiditis compared to controls, particularly in the categories of increased and definite CV risk (*p* = 0.002) (Figure 2). Moreover, participants with no or limited cardiovascular risk had significantly lower catestatin levels in comparison to those with increased or definite cardiovascular risk (9.33 ± 5.68 ng/mL vs. 16.76 ± 9.34 ng/mL, *p* = 0.011).

In Table 3, we evaluated the correlation of catestatin and AGE levels with various anthropometric and laboratory parameters in the total studied population. For catestatin, significant positive correlations were found with anti-TG (r = 0.405, *p* < 0.001), anti-TPO (r = 0.416, *p* < 0.001), and hsCRP (r = 0.204, *p* = 0.004. No significant correlations were observed with age, BMI, waist circumference, blood pressure, thyroid function tests (fT3, fT4), lipid profile, glucose, or creatinine. Regarding AGE levels, significant positive correlations were noted with age (r = 0.226, *p* = 0.001), BMI (r = 0.151, *p* = 0.033), and waist circumference (r = 0.166, *p* = 0.019). AGE levels also exhibited significant positive associations with anti-TG (r = 0.278, *p* < 0.001), anti-TPO (r = 0.397, *p* < 0.001), and approached significance with hsCRP (r = 0.138, *p* = 0.051). Non-significant correlations were observed with systolic and diastolic blood pressure, thyroid function tests, lipid profile, glucose and creatinine.

In Table 4, we examined the correlation of catestatin and AGE levels with various anthropometric and laboratory parameters in Hashimoto thyroiditis patients. Catestatin levels were significantly positively correlated with anti-TG (r = 0.353, *p* < 0.001), anti-TPO (r = 0.229, *p* = 0.024), and hsCRP (r = 0.277, *p* = 0.005). In contrast, no significant correlations were found between catestatin and age, BMI, waist circumference, blood pressure, thyroid function tests, or other biochemical markers (cholesterol levels, triglycerides, glucose, and creatinine). For AGE levels, significant positive correlations were observed with age (r = 0.247, *p* = 0.013), BMI (r = 0.200, *p* = 0.046), and waist circumference (r = 0.203, *p* = 0.042), as well as with anti-TG (r = 0.215, *p* = 0.032) and anti-TPO (r = 0.276, *p* = 0.005) antibodies. Non-significant correlations were noted with systolic and diastolic blood pressure, thyroid function tests, lipid profile, fasting glucose and creatinine.

Finally, a positive correlation was found between serum catestatin concentrations and AGE tissue levels, both in the total population (r = 0.489, *p* < 0.001) and in the Hashimoto group (r = 0.457, *p* < 0.001). Accordingly, the association remained significant even after adjustment for age, BMI, systolic blood pressure, hsCRP, TSH and anti-TPO serum concentrations in both study groups (Supplementary Tables S1 and S2).

## 4. Discussion

The present results indicate that serum catestatin concentrations and AGE tissue levels are elevated in patients with Hashimoto thyroiditis, even after adjustment for potential confounders. Moreover, both catestatin and AGEs seem to correlate with antibodies directed at thyroid antigens. Finally, in the Hashimoto population, serum catestatin concentrations were shown to be independently associated with hsCRP, AGEs, and anti-thyroid autoantibodies. To the best of our knowledge, this is the first report to assess serum catestatin levels in patients with Hashimoto’s thyroiditis showing its elevation.

Only two studies explored chromogranin A, a precursor of catestatin, levels in thyroid disorders. Polikar et al. found no difference between hypothyroid patients and controls, both before and after thyroid hormone replacement therapy [13]. On the other hand, a separate study showed that in hyperthyroid patients, pretreatment CgA levels are markedly higher than in controls, but they drop to levels similar to those of controls upon therapy [14]. Considering that our patient population was relatively concordant to that of the aforementioned study in hypothyroid patients, we could explain the increase in serum catestatin levels with CgA production and processing outside chromaffin cells. For instance, it is well established that CgA can be produced and processed to catestatin in various tissues such as the myocardium [15,16]. However, it is worth noting that both studies involved a limited number of patients with thyroid disorders (11 and 36, respectively), thereby restricting the ability to draw definitive conclusions based on their findings. Also, a study by Choi et al. found that catestatin Ser-364 allele may increase the risk for cardiovascular diseases in the Japanese population [17]. However, it remains unknown whether the observed increase in catestatin is part of a protective feedback mechanism in response to chronic low-grade inflammation and oxidative stress or if catestatin itself is a causative factor in the pathophysiology of these conditions. A common denominator that associates inflammatory disorders and increased catestatin concentrations seems to be endothelial dysfunction. It has been shown in various reports that Hashimoto’s thyroiditis is associated with endothelial dysfunction and atherosclerotic changes, thereby potentially increasing the risk of cardiovascular disease [18,19]. Specifically, patients with Hashimoto’s thyroiditis have been found to exhibit increased carotid intima-media thickness (cIMT) and impaired flow-mediated dilation (FMD), though not all studies have consistently demonstrated this association [20,21,22,23]. It has been proposed that low-grade chronic inflammation associated with Hashimoto’s thyroiditis leads to endothelial dysfunction and reduced nitric oxide availability through a COX-2-dependent pathway, independent of lipid metabolism changes [24]. Some authors have even suggested that thyroid autoantibodies may target the arterial wall and directly contribute to the development of atherosclerotic plaque [25,26]. However, a recent large-scale population-based study failed to demonstrate a significant association between autoantibodies and atherosclerotic outcomes [27].

Available data suggest that catestatin exerts multiple immunomodulatory functions: stimulation of mast cells to produce histamine and neutrophils to secrete lactotransferrin, reduction in pro-inflammatory cytokine release by macrophages, reduction in systemic inflammation by affecting the gut microbiome [28,29,30,31]. Immunomodulatory effects of catestatin especially pertain to the cardiovascular system. For instance, preclinical evidence suggests that catestatin can prevent atherosclerosis by suppressing tumor necrosis factor-α (TNF-α), vascular cell adhesion molecule-1 (VCAM-1), and intercellular adhesion molecule-1 (ICAM-1) in endothelial cells, as well as by inhibiting inflammatory responses and oxidized low-density lipoprotein-induced foam cell formation in human macrophages [32]. Given the independent positive association of catestatin with both hsCRP and tissue levels of AGEs, as well as thyroid antibodies, and consistent with available data, we hypothesize that elevated catestatin levels in Hashimoto’s thyroiditis reflect more pronounced endothelial dysfunction in these patients. Specifically, hsCRP is a well-established independent marker of endothelial dysfunction, while AGEs serve not only as markers of oxidative damage but also as promising predictors of endothelial dysfunction [33,34,35]. Overall, elevated AGEs, hsCRP and catestatin, alongside other factors, are in line with existing data suggesting increased cardiovascular risk in patients with Hashimoto’s thyroiditis [18,36]. Specifically, catestatin is a bioactive peptide known to influence cardiovascular function by modulating endothelial activity, inflammatory responses, and lipid metabolism—all critical factors in the development of atherosclerosis and cardiovascular disease. The elevated catestatin levels in our Hashimoto’s thyroiditis group suggest a potential mechanism for increased vascular complications, including endothelial dysfunction, heightened inflammation, and alterations in lipid metabolism. These findings support the hypothesis that individuals with Hashimoto’s thyroiditis may have a higher predisposition to vascular complications due to elevated catestatin levels. However, we acknowledge that while our study provides important initial evidence, further research is necessary to establish a direct causal relationship and to explore the potential of catestatin as a biomarker for cardiovascular risk assessment in this population group.

The results of the present study must be interpreted with caution, considering several study limitations. First, despite the study being adequately powered to demonstrate the primary outcome, increasing the number of participants, especially those of diverse ethnic backgrounds, would enhance the applicability of our results. Additionally, as only female patients were included, the results may not be generalizable to the male population. The cross-sectional nature of this research limits the establishment of causality, and multiple measurements would provide better insight into the dynamics of catestatin in Hashimoto’s thyroiditis. Accordingly, we are currently conducting a prospective study aimed at elucidating the effect of thyroid replacement therapy on catestatin and AGEs in these patients.

## 5. Conclusions 

In summary, for the first time, we established that serum catestatin concentrations are elevated in female patients with untreated Hashimoto’s thyroiditis. Independent associations between catestatin, AGE tissue levels, indices of inflammation, and thyroid autoantibodies suggest that catestatin may reflect the extent of endothelial dysfunction in these patients. Nonetheless, further larger-scale research is needed to establish the clinical relevance of these results and to elucidate the underlying mechanisms.

## Figures and Tables

**Table 1:** Baseline characteristics of the investigated population.

Parameter	Hashimoto Group (N = 100)	Control Group (N = 100)	*p* *
Age (years)	36.8 ± 12.6	35.2 ± 10.5	0.345
Body height (cm)	170.3 ± 6.6	171.5 ± 5.9	0.372
Body weight (kg)	68.2 ± 12.2	66.6 ± 10.6	0.326
Body mass index (kg/m^2^)	23.3 ± 4.9	22.7 ± 3.5	0.398
Waist circumference (cm)	79.2 ± 12.2	79.1 ± 10.5	0.978
Hip circumference (cm)	101.8 ± 9.1	100.5 ± 9.4	0.327
WHR	1.30 ± 0.09	1.28 ± 0.09	0.298
Systolic blood pressure (mmHg)	110.2 ± 15.5	112.4 ± 11.5	0.269
Diastolic blood pressure (mmHg)	68.1 ± 9.5	68.2 ± 7.7	0.935
Active smoking (yes)	37 (37.0)	32 (32.0)	0.458

Data are presented as mean ± standard deviation or whole number (%). Abbreviations: WHR—waist-to-hip ratio. * *t*-test for independent samples or chi-squared test.

**Table 2:** Laboratory findings of the investigated population.

Parameter	Hashimoto Group (N = 100)	Control Group (N = 100)	*p* *
TSH (mIU/L)	1.65 (1.4–2.9)	1.58 (1.22–2.12)	0.017
fT3 (pmol/L)	4.4 (4.3–4.85)	4.45 (4.2–4.8)	0.562
fT4 (pmol/L)	13.1 ± 1.81	13.7 ± 1.99	0.027
anti-TG (IU/mL)	119.1 (16.4–274.0)	9.2 (6.6–13.4)	<0.001
anti-TPO (IU/mL)	71.9 (21.7–243.0)	1.9 (1.3–4.1)	<0.001
hsCRP (mg/L)	2.1 (1.3–3.2)	1.2 (0.4–1.6)	<0.001
Total cholesterol (mmol/L)	5.28 ± 1.01	4.69 ± 1.42	0.088
HDL-cholesterol (mmol/L)	1.55 ± 0.31	1.53 ± 0.48	0.822
LDL-cholesterol (mmol/L)	3.24 ± 0.90	2.91 ± 1.12	0.025
Triglycerides (mmol/L)	1.44 ± 0.67	1.37 ± 1.08	0.573
Fasting glucose (mmol/L)	5.11 ± 0.49	4.93 ± 0.81	0.109
Creatinine (µmol/L)	69.3 ± 15.5	67.6 ± 10.7	0.358
Urea (mmol/L)	5.5 ± 1.77	5.31 ± 1.51	0.416

Data are presented as mean ± standard deviation or median (interquartile range). Abbreviations: TSH—thyroid stimulating hormone; fT3—free triiodothyronine; fT4—free thyroxine; anti-TG—thyroglobulin antibodies; anti-TPO—anti-thyroid peroxidase antibodies; hsCRP—high-sensitivity C-reactive protein; HDL—high-density lipoprotein; LDL—low-density lipoprotein. * *t*-test for independent samples or Mann–Whitney test, as appropriate.

**Table 3:** Correlation of catestatin and AGE levels in total population between selected anthropometric and laboratory parameters (N = 200).

Parameter	Catestatin *r* (*p* ^†^)	AGE *r* (*p* *)
Age (years)	0.052 (0.466)	**0.226 (0.001)**
Body mass index (kg/m^2^)	0.101 (0.155)	**0.151 (0.033)**
Waist circumference (cm)	0.053 (0.454)	**0.166 (0.019)**
Systolic blood pressure (mmHg)	0.003 (0.965)	−0.005 (0.945)
Diastolic blood pressure (mmHg)	0.051 (0.475)	0.057 (0.419)
TSH (mIU/L)	**0.182 (0.010)**	0.086 (0.225) ^†^
fT3 (pmol/L)	0.016 (0.822)	0.038 (0.591) **^†^**
fT4 (pmol/L)	−0.119 (0.0937)	−0.067 (0.344)
anti-TG (IU/mL)	**0.405 (<0.001)**	**0.278 (<0.001) ^†^**
anti-TPO (IU/mL)	**0.416 (<0.001)**	**0.397 (<0.001) ^†^**
hsCRP (mg/L)	**0.204 (0.004)**	0.138 (0.051) ^†^
Total cholesterol (mmol/L)	0.075 (0.289)	−0.003 (0.967)
HDL-cholesterol (mmol/L)	−0.043 (0.548)	−0.019 (0.792)
LDL-cholesterol (mmol/L)	0.056 (0.429)	0.068 (0.338)
Triglycerides (mmol/L)	0.011 (0.881)	−0.006 (0.933)
Fasting glucose (mmol/L)	0.064 (0.365)	0.043 (0.543)
Creatinine (µmol/L)	0.050 (0.478)	0.005 (0.940)

Abbreviations: AGE—Advanced Glycation End product; TSH—thyroid stimulating hormone; fT3—free triiodothyronine; fT4—free thyroxine; anti-TG—thyroglobulin antibodies; anti-TPO—anti-thyroid peroxidase antibodies; hsCRP—high-sensitivity C-reactive protein; HDL—high-density lipoprotein; LDL—low-density lipoprotein. Bolded values indicate statistical significance. * Pearson’s correlation test. † Spearman’s correlation test.

**Table 4:** Correlation of catestatin and AGE levels in Hashimoto group patients between selected anthropometric and laboratory parameters (N = 100).

Parameter	Catestatin *r* (*p* ^†^)	AGE *r* (*p* *)
Age (years)	0.151 (0.134)	**0.247 (0.013)**
Body mass index (kg/m^2^)	0.142 (0.159)	**0.200 (0.046)**
Waist circumference (cm)	0.124 (0.219)	**0.203 (0.042)**
Systolic blood pressure (mmHg)	−0.045 (0.661)	0.036 (0.721)
Diastolic blood pressure (mmHg)	0.071 (0.482)	0.093 (0.357)
TSH (mIU/L)	0.123 (0.223)	0.045 (0.660) ^†^
fT3 (pmol/L)	0.163 (0.105)	0.073 (0.469) ^†^
fT4 (pmol/L)	−0.090 (0.374)	−0.124 (0.218)
anti-TG (IU/mL)	**0.353 (<0.001)**	**0.215 (0.032) ^†^**
anti-TPO (IU/mL)	**0.229 (0.024)**	**0.276 (0.005) ^†^**
hsCRP (mg/L)	**0.277 (0.005)**	0.141 (0.163) ^†^
Total cholesterol (mmol/L)	0.040 (0.689)	−0.082 (0.416)
HDL-cholesterol (mmol/L)	0.092 (0.361)	0.092 (0.361)
LDL-cholesterol (mmol/L)	−0.029 (0.776)	0.010 (0.922)
Triglycerides (mmol/L)	−0.113 (0.263)	−0.003 (0.978)
Fasting glucose (mmol/L)	−0.007 (0.946)	−0.057 (0.576)
Creatinine (µmol/L)	0.089 (0.378)	−0.006 (0.952)

Abbreviations: AGE—Advanced Glycation End products; TSH—thyroid stimulating hormone; fT3—free triiodothyronine; fT4—free thyroxine; anti-TG—thyroglobulin antibodies; anti-TPO—anti-thyroid peroxidase antibodies; hsCRP—high-sensitivity C-reactive protein; HDL—high-density lipoprotein; LDL—low-density lipoprotein. Bolded values indicate statistical significance. * Pearson’s correlation test. † Spearman’s correlation test.

## Data Availability

The data presented in this study are available on request from the corresponding author. The data are not publicly available because some of the dataset will be used for further research.

## Supplementary Materials

The following supporting information can be downloaded at: https://www.mdpi.com/article/10.3390/biom15020169/s1. Table S1. Multiple linear regression model of independent predictors for serum catestatin levels in total investigated population (enter algorithm). Table S2. Multiple linear regression model of independent predictors for serum catestatin levels in total investigated population (forward algorithm). Table S3. Multiple linear regression model of independent predictors for AGE levels in total investigated population (enter algorithm). Table S4. Multiple linear regression model of independent predictors for AGE levels in total investigated population (forward algorithm).
