# Supplementary material for: Catestatin and Advanced Glycation End-Products: Potential Indicators of Cardiovascular Risk in Hashimoto’s Thyroiditis
Source: Biomolecules. 2025 Jan 23;15(2):169. doi: 10.3390/biom15020169 (PMC11852764; doi:10.3390/biom15020169)
Supplement: Supplementary file 1 [file biomolecules-15-00169-s001.zip › biomolecules-3267636-supplementary.pdf]

Supplementary Materials:

Supplementary Table S1. Multiple linear regression model of independent predictors for serum catestatin levels in total investigated population (enter algorithm).

**Variable**	**β***	**SE†**	**P**
Age (years)	0.054	0.040	0.483
Hashimoto thyroiditis	1.819	0.830	0.030
Body mass index (kg/m^2)	0.009	0.090	0.928
Systolic blood pressure (mmHg)	-0.026	0.032	0.410
TSH (mIU/L)	0.007	0.05	0.888
anti-TPO (IU/mL)	0.182	0.001	0.011
hsCRP (mg/L)	0.809	0.261	0.002
AGEs	5.400	1.745	0.002

Abbreviations: AGEs- Advanced Glycation End products; hsCRP- high sensitive C-reactive protein
* standardized coefficient β
† standard error

Supplementary Table S2. Multiple linear regression model of independent predictors for serum catestatin levels in total investigated population (forward algorithm).

**Variable**	**β***	**SE†**	**P**
Age (years)	ns	ns	ns
Hashimoto thyroiditis	1.802	0.758	0.018
Body mass index (kg/m^2)	ns‡	ns‡	ns‡
Systolic blood pressure (mmHg)	ns‡	ns‡	ns‡
TSH (mIU/L)	ns‡	ns‡	ns‡
anti-TPO (IU/mL)	0.003	0.001	0.023
hsCRP (mg/L)	0.782	0.242	0.001
AGEs	3.949	0.628	<0.001

Abbreviations: AGEs- Advanced Glycation End products; hsCRP- high sensitive C-reactive protein
* standardized coefficient β
† standard error
‡ non-significant

Supplementary Table S3. Multiple linear regression model of independent predictors for AGE levels in total investigated population (enter algorithm).

**Variable**	**β***	**SE†**	**P**
Age (years)	0.147	0.004	0.036
Hashimoto thyroiditis	1.419	0.597	0.019
Body mass index (kg/m^2)	0.062	0.009	0.387
Systolic blood pressure (mmHg)	-0.031	0.003	0.650
TSH (mIU/L)	0.017	0.040	0.791
anti-TPO (IU/mL)	0.128	0.001	0.021
hsCRP (mg/L)	1.090	0.240	<0.001
Catestatin (ng/mL)	0.038	0.006	<0.001

Abbreviations: AGEs- Advanced Glycation End products; hsCRP- high sensitive C-reactive protein
* standardized coefficient β
† standard error

Supplementary Table S4. Multiple linear regression model of independent predictors for AGE levels in total investigated population (forward algorithm).

**Variable**	**β***	**SE†**	**P**
Age (years)	0.007	0.003	0.027
Hashimoto thyroiditis	0.226	0.07	0.003
Body mass index (kg/m^2)	ns‡	ns‡	ns‡
Systolic blood pressure (mmHg)	ns‡	ns‡	ns‡
TSH (mIU/L)	ns‡	ns‡	ns‡
anti-TPO (IU/mL)	0.0002	0.001	0.046
hsCRP (mg/L)	0.078	0.026	0.003
Catestatin (ng/mL)	0.039	0.006	<0.001

Abbreviations: AGEs- Advanced Glycation End products; hsCRP- high sensitive C-reactive protein
* standardized coefficient β
† standard error
‡ non-significant
